# Supplementary material for: Ultra-large library screening with an evolutionary algorithm in Rosetta (REvoLd)
Source: Commun Chem. 2025 Nov 7;8:335. doi: 10.1038/s42004-025-01758-x (PMC12594993; doi:10.1038/s42004-025-01758-x)
Supplement: Supplementary file 2 — Supplementary Information [file 42004_2025_1758_MOESM2_ESM.pdf]

Supplementary Information for Ultra-Large Library Screening with an Evolutionary Algorithm in Rosetta (REvoLd)

Paul Eisenhuth[1,2*], Fabian Liessmann[1], Rocco Moretti[3], Jens Meiler[1-4]

[1]Institute for Drug Discovery, Leipzig University, Leipzig, 04103, Germany.
[2]Center for Scalable Data Analytics and Artificial Intelligence (ScaDS.AI) Dresden/Leipzig, Leipzig University, Leipzig, 04105, Germany.
[3]Center for Structural Biology, Vanderbilt University, Nashville, 37235, TN, USA.
[4]Department of Chemistry, Vanderbilt University, Nashville, 37235, TN, USA.

*Corresponding author(s). E-mail(s): eisenhuth@cs.uni-leipzig.de;

Table S1 Different hyperparameters tested in the artificial benchmark

Enrichment	Generations	Population Size	Initial Size	Tournament	Protocol
16.14	30	50	100	5	Vanilla
15.05	15	50	100	5	Vanilla
17.01	50	50	100	5	Vanilla
16.03	75	50	100	5	Vanilla
17.35	100	50	100	5	Vanilla
15.71	30	50	50	5	Vanilla
18.52	30	50	150	5	Vanilla
16.83	30	50	200	5	Vanilla
13.65	30	20	100	5	Vanilla
16.77	30	30	100	5	Vanilla
17.85	30	40	100	5	Vanilla
15.58	30	60	100	5	Vanilla
13.70	15	30	50	5	Vanilla
9.64	15	30	50	5	Vanilla low rep
13.29	10	50	100	5	Vanilla high rep
17.56	30	50	100	5	Exploration
18.43	30	50	200	5	Exploration
26.03	30	50	200	10	Exploration
27.43	30	50	200	15	Exploration
**30.41**	**30**	**50**	**200**	**15**	**Explore-Crossover**
24.81	15	50	200	15	Explore-Crossover
34.39	50	50	200	15	Explore-Crossover
37.29[1]	100	50	200	15	Explore-Crossover
38.61[2]	150	50	200	15	Explore-Crossover
28.45[2]	400	50	200	15	Explore-Crossover

The reported enrichment is averaged over 20 runs if not stated otherwise. The bold line are the settings we used for our final protocol.

[1]Only five runs were conducted

[2]Only a single run was conducted

1 Hyperparameter optimization

Table S1 shows all settings we tested for the hyperparameter optimization on the artificial benchmark explained in the main paper. Every setting was tested with 20 independent runs and the average enrichment is reported if not stated otherwise. Tournament selector was used for all protocols as main selector, but with varying tournament sizes. A larger tournament size means a smaller chance for less optimal molecules to remain in the population. Initially, we tested the impacts of number of generations, the size of population passing between generations and the initial population size of random molecules. The reported enrichment factor is over the empirically known hit rate of the artificial test set, which is $0.001\frac{hits}{molecule}$, since we selected the best 1,000 molecules from the one million set as hits. Whilst this list is far from covering all possible combinations, we could observe a strong increase in enrichment and found a set of well performing and reliable parameters.

The mentioned vanilla protocol was our initial protocol focusing on optimization. No individuals were ever removed from the reproduction candidate pool which allowed already good scoring molecules to reproduce several times. The protocol passed the

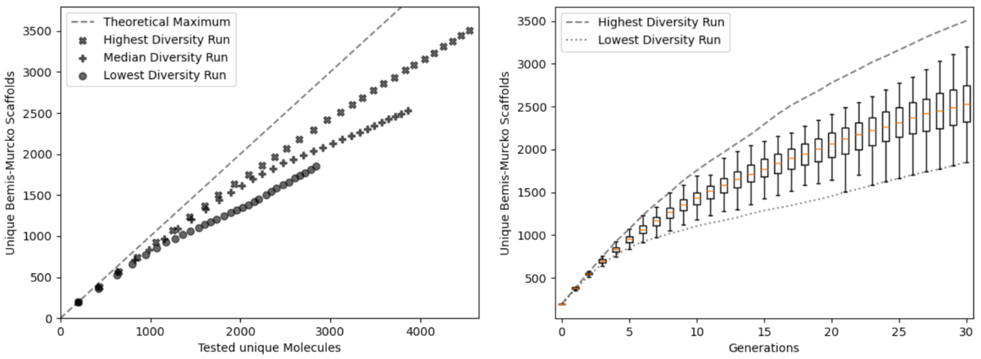

Fig. S1 Scaffold Diversity Analysis *Left*: Correlation between the number of unique tested molecules and number of unique Bermis-Murcko scaffold. We plotted the runs with lowest, median and highest number of unique scaffolds in the final generation from all runs targeting all proteins. Each point represents the ration between unique molecules and unique scaffolds at the end of each generation. *Right*: The distribution of the amount of discovered unique scaffolds over all runs per generation. Outliers are not shown, but approximated through the two runs reaching the highest and lowest count of unique scaffolds in the final generation.

15 best molecules of each generation unchanged into the next generation, produced 30 new molecules through small mutations with a bias towards changes in reagents instead of reactions and 30 more molecules through crossover. We made two slight changes to it by decreasing the reproduction numbers (Vanilla low rep) and increasing them (Vanilla high rep). Next, we came up with the protocol mentioned in the methods section, but with only 30 molecules from each crossover instead of 60. This is named Exploration in table S1. Finally, we increased the amount of crossover to 60 which is labeled as Explore-Crossover. Although the same protocol has higher hit rates if run for more generations, we found that this would tremendously increase the runtime. Therefore, we opted for the settings highlighted in bold.

2 Molecular diversity during runs

In the main paper we stated that REvoLd reliably samples new molecular scaffolds for all five target proteins. Figure S1 shows on the left side that the number of unique scaffolds and the number of unique molecules are linearly related. This indicates that the deployed sampling approaches are successfully exploring chemical space. We plotted the runs with lowest, median and highest number of unique scaffolds in the final generation from all runs targeting all proteins. There was no observable difference between the targets. Additionally, the right side shows that the number of unique scaffolds increases quickly during the first generation, but starts to slow down around generation 10. This is line with our findings from the hyperparameter optimization where we saw a decrease of discovery rates during later generations.

Next, we investigated how much the sampled molecules depend on the run starting population. Therefore, we calculated the maximum Tanimoto similarity to a molecule in the start population for each molecule in all following generations and reported the

mean for each run and each generation. The distribution of these mean similarities are shown as box plots. Figure S2 shows that all runs develop away from the starting population but maintain some similarities. Here we show again separate figures for each target due to slightly different observations. The difference between the final set of molecules and the starting population is further highlighted by the four example molecules in the top right corner of figure S2. We selected the run with the lowest diversity between start and final population (which targeted Tyrosyl) and binned both population by molecular size into three groups. The displayed molecules are scaffolds of the best scoring entries from the middle and large bin. We omitted the small size bin, as its entry scaffolds were very fragment-like. To clarify, the displayed similarities are between molecules, only the four examples are scaffolds.

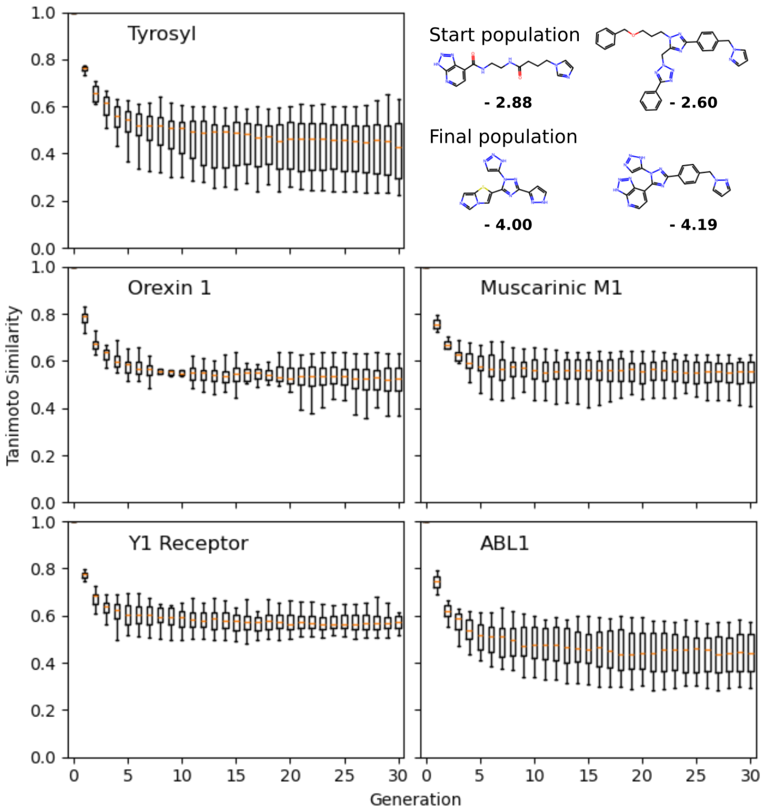

Fig. S2 Distribution of mean similarities between each generation molecules and their most similar predecessor in the initial population Between all five protein targets a clear trend can be observed, where with each generation the mean moves away from the starting population whilst maintaining some similarities. The right corner shows best scoring molecules from start and final population from a run against Tyrosryl which reported the overall lowest diversity of all runs. The molecules are selected based on their sizes and scores.

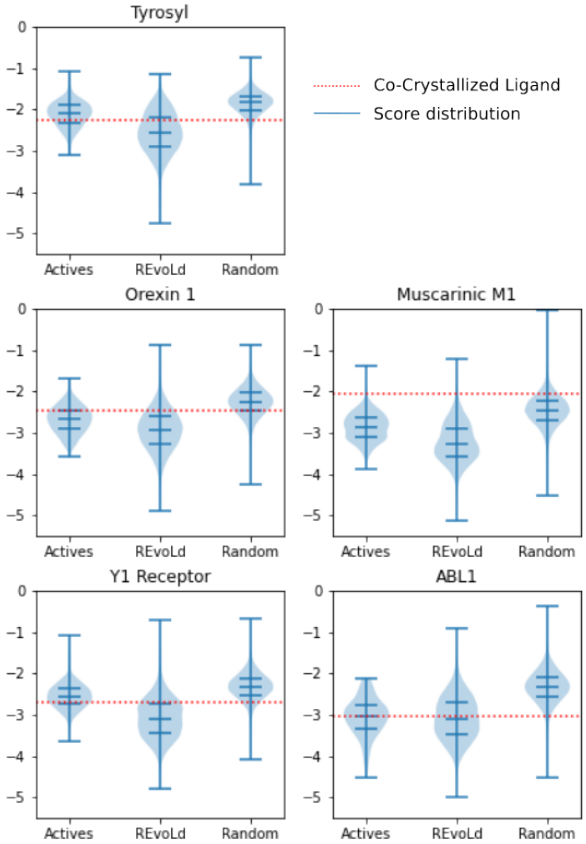

Fig. S3 **Score distribution** for all five targets used in the main paper for their known actives, all REvoLd results, the random sample and the ligand co-crystallized with the used protein structure. The violin plots show the density for given scores. The blue horizontal lines are from top to bottom the highest reported score, the first quantile, the second quantile (or median), the third quantile and lowest reported score. The bulk of the known actives report more negatives scores than the random sample, indicating that *lid_root2* can be used to enrich for activity. This is further supported by the co-crystallized score which is in all but one case within the known actives and below most of the random sample. REvoLd reports a much more negative score distribution, highlighting its optimization capabilities.

3 Score distribution of co-crystallized ligand, known actives, random samples and REvoLd

We compared the results from docking known actives, the co-crystallized ligands, and the random samples as mentioned in the main paper. Figure S3 shows that in all cases the average of the actives has a lower energy score than the random compounds, which indicates that optimizing over *lid_root2* is a valid method of finding actives. In all but one case (M1) the crystallized ligand is within the bulk of the active compounds, making it a decent proxy for the reference level of an active compound. However, the score is not a perfect classifier since there is still a large overlap between the random

sample and known binders. This can be due to actual unknown binders being included in the random sample or artifacts from the in-silico docking. Nonetheless, it shows that RosettaLigand and the *lid_root2* normalization assign more negative scores to known binders and therefore enrich binding in sets of molecules.

4 Comparison of different normalization methods

We tested our normalization method with four different n, where the penalty for high numbers of heavy atoms decreases with increasing n. To do so, we docked known actives for four of the five targets mentioned in the main paper against their respective proteins. These docking scores where then normalized and plotted against the number of heavy atoms (Figure S4). The results confirm the negative correlation between unnormalized scores and the number of heavy atoms as well as our expected impact of n. Whilst $n = 3$ and $n = 4$ seem to be more successful in negating the correlation, we opted to use $n = 2$. Higher n can sometimes reintroduce the negative correlation, for example for the orexin receptor. Additionally, we found a slight positive correlation desirable to actively limit the size of ligands during evolutionary optimization. However, $n = 1$ is too strict and favors fragment-like molecules. Figure S5 shows the lowest scoring molecules from the known actives for their respective targets and each normalization method. This reaffirms our findings that $n = 3$ and $n = 4$ are very similar to unnormalized scores and that $n = 1$ can result in fragment-like molecules.

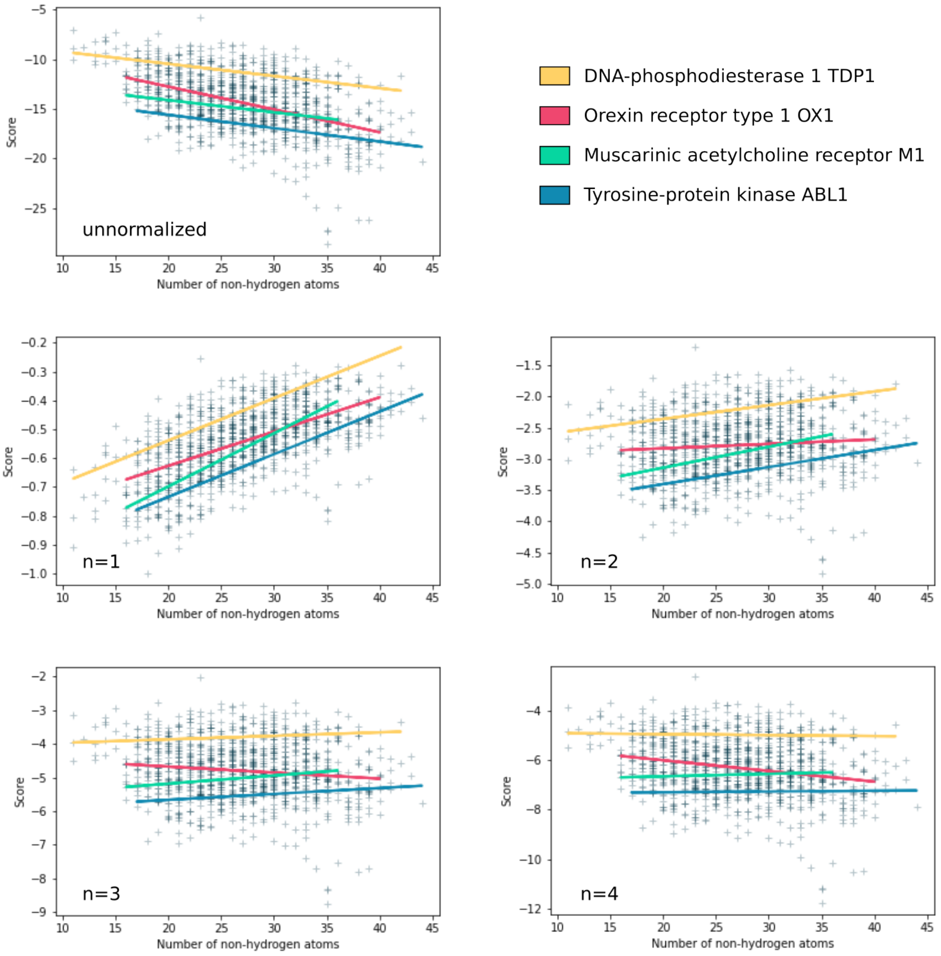

Fig. S4 Quantitative comparison of all four investigated normalization method across four targets with unnormalized scores The same known actives as in the main paper were docked and their interface scores in Rosetta energy units plotted against the number of heavy atoms. The normalization methods are mentioned in the methods section. Results of different targets are plotted all together as grey crosses, but their linear regressions are plotted individually. The unnormalized scores clearly shows the expected negative correlation between score and number of heavy atoms. Normalization with $n = 1$ on the other hand has a strong positive correlation. This correlation becomes less with increasing n until $n = 4$ starts to show a negative correlation again.

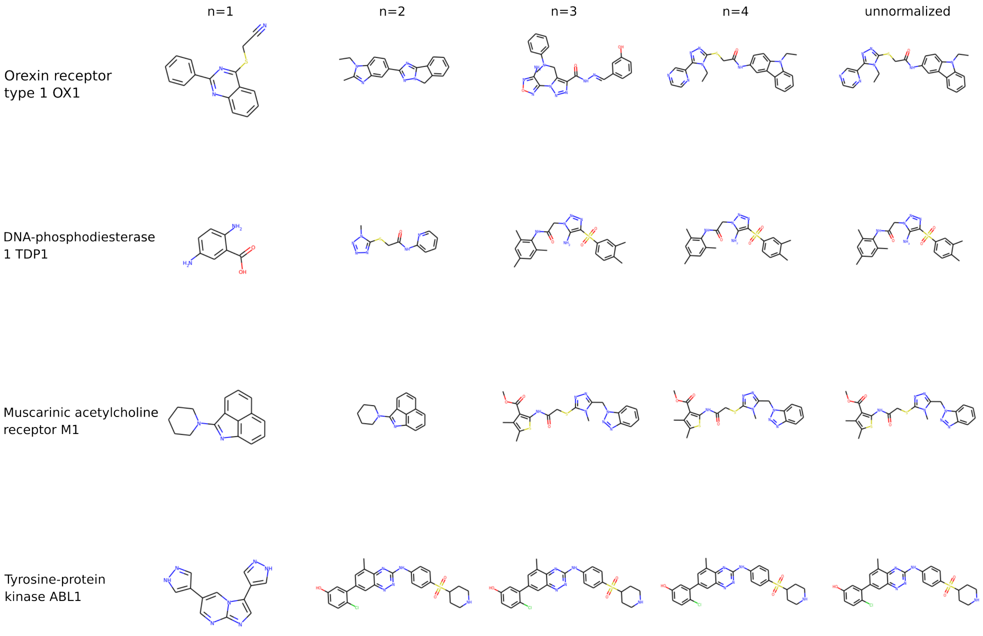

Fig. S5 Qualitative comparison of all four investigated normalization method across four targets with unnormalized scores The same known actives as in the main paper were docked and the lowest (and therefore best) scoring compounds are shown for each target and normalization method. The normalization methods are mentioned in main paper methods section. The best molecules increase in size with less strong normalization for all tested targets. $n = 4$ reports the same molecule as the unnormalized scoring. This is also true for $n = 3$ except for OX1. Additionally, ABL1 still has the same best molecule for $n = 3$.
